# Assessment of Knowledge, Attitudes, and Practices on Water, Sanitation, and Hygiene in Some Selected LGAs in Kaduna State, Northwestern Nigeria

**DOI:** 10.1155/2020/6532512

**Published:** 2020-08-31

**Authors:** M. K. C. Sridhar, O. T. Okareh, M. Mustapha

**Affiliations:** ^1^Department of Environmental Health Sciences, Faculty of Public Health, College of Medicine, University of Ibadan, Ibadan, Nigeria; ^2^Department of Agricultural and Environmental Resources Engineering, Faculty of Engineering, University of Maiduguri, Maiduguri, Nigeria

## Abstract

Access to safe water, sanitation, and hygiene (WASH) facilities is a basic necessity for human livelihood, survival, and well-being. Adequate WASH facilities provision is a critical issue to most developing countries around the world including Nigeria. Knowledge, attitudes, and practices regarding WASH are integral to effective and sustainable WASH facilities provision. This study assessed the level of knowledge, behavior, and practices towards water, sanitation, and hygiene in Kaduna state, Nigeria, with a view to ensuring sustainable WASH facilities intervention in the region. Data collection tools included spot check observation and questionnaire involving 854 participants, selected from five local government areas (LGAs): Chikun, Kajuru, Soba, Kachia, and Zango Kataf. From the results, major drinking water sources were surface waters (52.5%) and unprotected hand dug wells (44.8%); only 46.2% treated their water supply and few (16.6%) used chlorination method. Pit latrine toilets were the major (76.5%) excreta disposal means, and open defecation practices were widespread (41.4%). Level of personal and environmental hygiene understanding was fairly good in all the local government areas, and 65.4% claimed to use water and soap for washing hands after defecation. Incidence of water related diseases is generally low in the area. Despite the commendable findings in the study areas, communities are still at risk due to lack of safe water supply and poor practices of home treatment and excreta disposal. Therefore provision of WASH facilities and WASH education is fundamental for ensuring public health in the study area.

## 1. Introduction 

Access to safe water, sanitation, and hygiene (WASH) facilities is considered a basic human necessity for survival and well-being [1], without these basic needs, the health conditions of millions of people especially children are at risk [2]. However, 2.3 billion and 844 million people across the globe lack access to basic drinking water and sanitation facilities, respectively [3], causing 842,000 deaths every year [4], which is undoubtedly a major public health concern. WASH services are considered means of contacting and at the same time preventing diseases [5]. It has been estimated that overall 9% of the global burden of disease could be prevented through improvement in adequate WASH facilities [6, 7]. Children are one of the most vulnerable groups affected by lack of water, sanitation, and hygiene facilities [2]. In developing countries, the high mortality rate resulting from diarrheal among children under the age of five was majorly due to WASH challenges [2, 5].

The provision of safe WASH facilities has been greatly influential on people's health status and livelihood; however, the availability of these facilities remains critical in Nigeria especially in the rural areas [8–12]; a large percentage of rural communities in Nigeria live without access to safe WASH facilities [13]. The situation has thus subjected the communities to the utilization of water from rivers, ponds, and streams for drinking and domestic activities [14] and to the practice of open defecation [15] which consequently has often led to deaths, illnesses, and spread of waterborne diseases [16–18]. The few improved water facilities from boreholes and wells with hand pumps available are largely insufficient; women and children mostly travel far distances to access water, which is energy and time consuming, thus affecting children's education and women's household and economic productivity [19]. On the other hand, hygiene facilities such as excreta disposal (toilets) have also been inadequate for usage at community households and public places such as schools [20], markets [21], and even hospitals [22], which left people with no alternative but to defecate openly and sometimes in and around water sources [23] with no use of soap or any cleaning agents for protection [24]. Moreover, toilet facilities available were poorly maintained and mostly shared among numerous people with no consideration of gender segregation and women integrity [21]. However, government failures have led to the intervention by organizations such as UNICEF and WHO for aid through their programs known as *WASH*. UNICEF's WASH team works in over 100 countries globally to provide water and sanitation facilities [25].

Knowledge, attitudes, and practices (KAP) associated with WASH are of pertinent concern towards sustainable and effective implementation of WASH programs in communities [26]. KAP regarding WASH are contributing factors to waterborne disease prevalence in communities; poor WASH knowledge leads to unhygienic practices and poor attitudes which pollute water and spread illness [27, 28]. Such inadequate WASH knowledge leads to wrong perception of quality of water resulting in large dependence on surface waters for drinking [14, 29], open defection practices being perceived normal and commonly practiced, minimal household water purification practices to prevent diseases [30, 31], and poor water collection and storage behaviors contaminating water and causing illnesses [32–34]. Household and environmental hygiene also tend to be poor, and children stool is often overlooked and perceived harmless in sanitation programs, hence increasing the risk of disease transmission [35–37], all due to limited WASH understanding and poor attitudes and practices towards WASH. Therefore, there is a need to provide hygiene education programs and increased awareness towards promoting good WASH practices and ensuring good public health in the communities. In Nigeria, it is expected that there is currently a dearth of data on the status of WASH; thus, it is increasingly becoming difficult to plan any meaningful WASH program to improve health and well-being. Therefore, this study aims at assessing the knowledge, attitudes, and practices related to WASH in 5 local government areas in Kaduna state, Nigeria, with a view to providing reliable and sufficient data for effective implementation of WASH programs and ensuring good public health. The KAP survey is a follow-up of an earlier nationwide survey on water, sanitation, and hygiene carried out by UNICEF, Nigeria, during the years 2007–2009. The study objectives are to assess the demographic characteristics of the study areas relevant to the survey, the various water sources for drinking and domestic activities, home water treatment methods and practices, water collection and storage attitudes and practices, knowledge of quality drinking water, excreta disposal systems used and related perceptions, personal and environmental hygiene, and water related diseases relevant to the study.

## 2. Materials and Methods

### 2.1. Study Area

**Figure 1:**
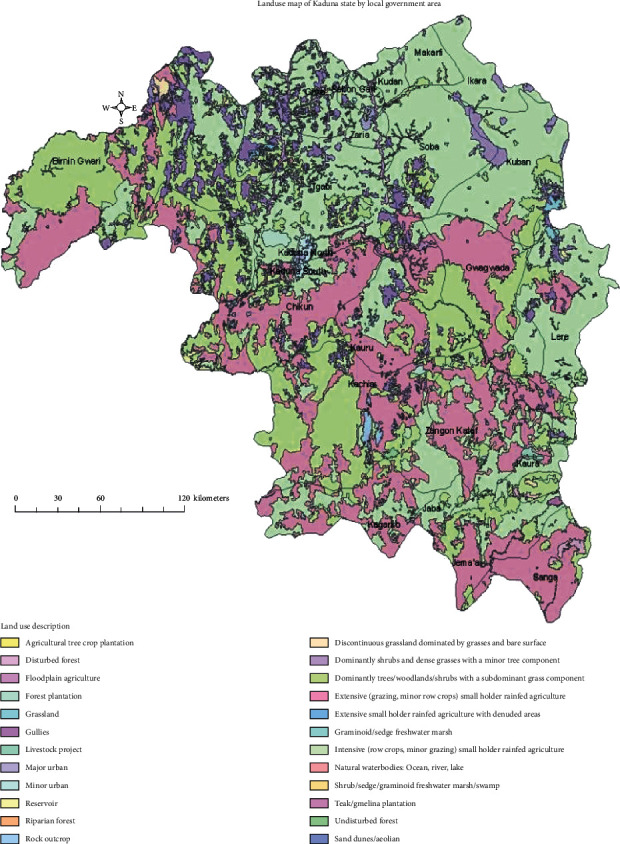
Map of Kaduna state with various LGAs.

Figure 1 shows the map of Kaduna state. It is located on the southern end of the high plains of Northern Nigeria. The state is situated between longitudes 06.00 and 09.00 east of the Greenwich Meridian, and between latitudes 09.00 and 11.00 north of the Equator. The major cities of Kaduna state are Kaduna town, Zaria, and Kafanchan. Other cities include Kachia, Zonkwa, Saminaka, Birnin Gwari, Makarfi, Ikara, Giwa, Zango Kataf, and Kagoro. Kaduna is the capital of the state and consists of two LGAs (Kaduna North and Kaduna South). The state shares common borders with Zamfara, Katsina, and Kano to the north; Niger to the west; Nasarawa to the south; Bauchi and Plateau to the east; and the Federal Capital Territory, Abuja, to the southwest. Kaduna state has an area of 46,053 square kilometers. The current projected population of Kaduna state (based on the 2016 population census) is estimated at 8,252,366. For conducting the KAP survey, 5 LGAs (Chikun, Kajuru, Soba, Kachia, and Z/Kataf) were selected from 23 LGAs (Figure 1). From each LGA, one community was picked up randomly for the KAP survey. The communities are Gwagwada (Chikun LGA), M/Kajuru (Kajuru LGA), Soba (Soba LGA), Kachia (Gumel) (Kachia LGA), and Gukwu (Z/kataf LGA). 

### 2.2. Sample Size and Survey Procedure

Scientific steps were followed to ensure the technical appropriateness of the survey sample size and sampling procedure. The following formula was used to determine the sample size [38]:

(1) $$\begin{array}{c} n=\frac{D^{*}\cdot Z_{\left(1-\alpha \right)/2}^{2}p\left(1-p\right)N}{d^{2}\left(N-1\right)+Z_{\left(1-\alpha \right)/2}^{2}p\left(1-p\right)}, \end{array}$$

where *n* is the sample size estimate, *D* is the design effect, *p* is the proportion practicing hand washing as a hygiene practice, *Z*_1−*α*/2_ is the standard score corresponding to 95% confidence level, *N* is the total population, and *d* is the degree of precision.

Using hand washing as a proxy for the indicators, *p* was chosen to be 50% (the value that will yield the largest sample size). The design effect was estimated at 2 because of the clustering in the target population, and the level of precision was set to 0.05. The total population of Kaduna state was obtained from the official gazette of the Federal Republic of Nigeria for the 2016 population census and used as N. After the application of the above formula, the estimate obtained for Kaduna state was adjusted for a nonresponse rate of 10%.

A three-stage sampling procedure was adopted for selection of respondents. In Kaduna state, the IYS (International Year of Sanitation) LGAs were 5 (Table 1). The first stage was a random selection of 40% of the IYS LGAs in each of the state LGAs. The NPC population estimates of the selected LGAs were obtained from the 2016 census [39]. The proportion of the total population constituted by each LGA was computed. Sample sizes for each LGA were then proportionately allocated to the selected communities.

The second stage involved the random selection of 40% of the IYS communities in each of the selected LGA. The sample sizes allocated to each LGA were equally allocated to the selected communities. Table 1 shows the breakdown of sample sizes for the state and selected LGAs and communities. The last stage was the random selection of street blocks (as clusters) based on the map of the selected community. Household heads (or representatives) were interviewed in selected clusters.

### 2.3. Data Collection and Analysis

The study is a cross-sectional field survey involving the use of structured questionnaire and field observation. A total of 854 questionnaires were administered and retrieved. The number was distributed across the LGAs as follows: Chikun: 236 (27.6%); Kachia: 156 (18.3%); Kajuru: 71 (8.3%); Soba: 188 (22.0%); and Zango/K: 203 (23.8%). The study variables include sociodemographic characteristics, water sources, and KAP regarding household water treatment, water collection and storage systems, excreta disposal, and household and environmental hygiene. In order to support the questionnaire data, observational checklists were used to capture and ensure the practices of household compound cleanliness, excreta disposal, and storage systems. Data collection was performed and supervised by 3 trained and experienced environmental and public public health professionals with assistant from various trained field staff members and researchers in Kaduna state. The survey was closely monitored for data quality assurance. Results of the surveys were entered into EpiData and analyzed.

## 3. Results

### 3.1. Background Characteristics of the Study Population

Table 2 shows the background characteristics of the survey respondents of which the majority (84.5%) were males. About 81.8% of them have been residents for more than 10 years. Some 47.6% were aged 30–50 years, and about 88.8% were married. Some 80.2% reported that they have been to school. However, 36.1% of them had Quranic education, 20.4% primary schooling, and 24.4% secondary schooling, while 14.0% had postsecondary education. Hausa accounted for 39.7% of the ethnic groups; others accounted for 58.4% in the selected LGAs. Christianity is the most common religion of the respondents (63.4%) followed by Islam (32.6%). Major occupations were farming (50.9%), civil service (17.1%), and self-employed (7.9%), and unemployment stood at 3.4%.

### 3.2. Sources of Water for Drinking and Other Activities in the Communities

As shown in Table 3, across the 5 LGAs, sources of drinking water include surface waters (52.5%), unprotected hand dug wells (44.8%), and protected hand dug wells (36.2%). Rain water harvesting was practiced by 52.5% of the communities. Kachia and Zango/K collect rain water to a large extent, 85.8% and 63.9%, respectively. These communities also utilize surface waters to a great extent, 89.0% and 95.1%, respectively. During dry season, unprotected hand dug wells (36.2%) and surface waters (51.9%) were the main sources. However, some still sought additional water from vendors. The sources of water for other domestic purposes were as follows: surface waters (47.8%), unprotected hand dug wells (39.16%), and protected hand dug wells (34.9%).

### 3.3. Water Treatment Methods Used and Communities Level of Practice

**Figure 2:**
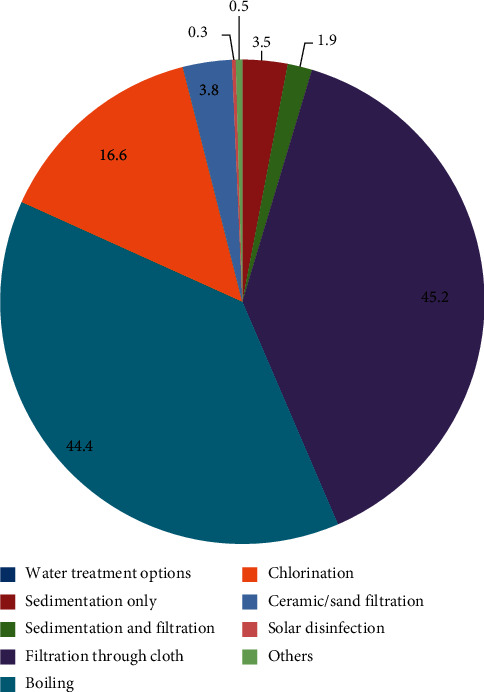
Household water treatment methods used.

About 46.2% of the respondents claimed to have treated their water supply (Table 4). The most common method was filtration through cloth (45.2%). Other methods mentioned were boiling (44.4%) and chlorination, though very few (16.6%) as shown in Figure 2. There is frequency of household water treatment in all the LGAs as 43.3% indicated that they treated water that day, 22.6% the previous day, 15.8% less than one week ago, 3.3% less than a month ago, and 5.7% more than a month ago, and very few (9.3%) could not remember the last time they treated water.

### 3.4. Household Drinking Water Storage Practices, Handling Attitudes, and Knowledge of Quality Drinking Water

The facilities used for storing drinking water were mostly covered clay pots (54.1%) and covered plastic containers (48.4%) (Table 5). Open containers were also common among 15.1% of the respondents. Plastic buckets with taps were used only in Kachia by 24.5% of the respondents. Items used in fetching drinking water from the storage facility included cup with handle (84.9%), cup without handle (4.9%), calabash (8.3%), and bowl (2.0%). These items were common in all the LGAs studied. These items were either kept on the storage container (59.4%), kept in a basket or shelf (22.5%), or hung (12.6%). Cleaning of the storage facilities was done daily by 72.6%, weekly by 15.0%, and when dirty by 11.5%. Again, the frequency of cleaning varied widely across the LGAs. When respondents were asked about the qualities of safe drinking water, there were responses such as visually clear (65.4%), free from germs (40.0%), odourless (36.0%), and sweet taste (30.0%). These views were consistent among the LGAs.

### 3.5. Excreta Disposal, Preferences, and Affordability

#### 3.5.1. Excreta Disposal Methods and Practices in the Study Area

The use of traditional pit latrine was a common practice in all the 5 LGAs as 76.5% claimed usage (Table 6). However, about 41.4% still practice open defecation. Kachia and Kajuru had large number of traditional pit toilets. Improved pit toilets and VIP toilets were also found in Kachia (47.1%) and Kajuru (23.3%). In the households, 65.7% use traditional pit latrines, 5.9% use improved pit toilets, and 22.8% practice open defection. People use these facilities because they are cheap (25.7%) or easy to maintain (31.9%) or because they cannot afford to build a better one (30.5%). Among those practicing open defecation, 90.3% were willing to stop and start using traditional pit latrine (37.9%) and improved pit toilets (27.1%). Those who were not willing to stop open defecation cited lack of money as being the reason.

In the communities, it was common for under-5 children to defecate around the house (25.4%), in the toilet (28.32%), and in the potty/chamber pot (24.7%). Defecation around the house is most common in Zango (47.8%). The use of potty/chamber pot was more frequent in Kajuru (37.0%) and Chikun (34.0%). After children defecation, the feces were dropped into a toilet facility (63.0%), thrown into the bush (16.3%), or eaten by dogs (10.4%). Dropping of children feces into toilet facility was generally a common practice across the LGAs as shown in Table 7.

#### 3.5.2. Perception of a Good Toilet, Type of Toilet, and Preferred Ownership by the Population

The information on how the respondents perceive a good toilet, the type of toilet, and preferred ownership is displayed in Table 8. In terms of perception, the respondents were of the opinion that privacy (46.4%), disease prevention (46.0%), and safety (33.1%) mean a good toilet. Most (70.9%) of the respondents preferred private toilet, some preferred compound toilet (22.4%), and very few opted for communal toilets (6.8%). Flush toilet (56.5%) and traditional pit toilet (25.4%) are the most preferred types in the selected LGAs followed by VIP (14.6%) and San Plat (3.5%) types. About 73.8% could afford the preferred toilet type, and only 7.8% are willing to contribute towards the preferred toilet.

### 3.6. Personal, Household, and Environmental Hygiene Practices of the Respondents

#### 3.6.1. Personal Hygiene

The respondents used soap for washing clothes (77.5%), taking bath (85.4%), bathing children (49.4%), and washing hands after defecation (31.3%) as shown in Table 9. When asked about when is it important to wash hands, there was a consensus among the respondents from the LGAs that hands should be washed before meal (85.1%), after meal (73.5%), after defecation (46.8%), and after cleaning children feces (13.5%). After defecation, hand washing is practiced the most in Kachia (76.8%) followed by Zango (53.7%). Items used for hand washing include soap with water (65.4%), water only (21.6%), and sand and water (8.8%). A sizable number understood that personal hygiene means bathing (87.5%), washing of clothes (63.0%), cutting of hair (46.4%), and cutting of nails (46.9%). The knowledge was comparable among the communities.

#### 3.6.2. Household and Environmental Hygiene

Sweeping of the house (90.6%), cleaning of kitchen (42.8%), proper disposal of waste water (35.5%), cleaning of toilets (46.6%), and proper disposal of solid waste (30.6%) regularly are practices referred to as household and environmental hygiene by the respondents. This understanding of household/environmental hygiene is fairly uniform in all the LGAs. When asked how often they clean their compounds, respondents said once daily (54.6%), every other day (8.7%), and only when dirty (10.4%). Taking refuse to the dump sites (56.5%) is the most common way of household waste disposal, while other practices are open dumping (26.2%) and burning 26.1%. With respect to animal waste, the majority (79.9%) take it to the farms while 6.4% dump it openly. Presence of stagnant water around water points was reported by only 23.3%, and this was the highest in Kajuru (41.1%) and the lowest in Chikun (2.9%). In order to prevent water stagnation, 81.0% said they would clear it, while 3.0% said they would divert it to farms. There was however a consensus (94.5%) that community members should be responsible for prevention of stagnated water.

### 3.7. Water Related Diseases Perceived in the Households and Communities

There were reports of epidemics of water related diseases within the last year by 40.9% of all respondents. The common diseases are malaria (88.6%), typhoid (56.5%), measles (51.8%), and diarrhea/dysentery (33.1%) as shown in Table 10. These are prevalent in all the LGAs. Diseases of great concern are typhoid and diarrhea which were relatively more in Kachia and Zango LGAs. Yellow fever was reported to be high in Kachia (60.6%), and no Guinea worm was reported in any of the LGAs.

### 3.8. Structured (Spot Check) Observations Findings

The results of the spot checks are summarized in Table 11. Presence of feces was reported around the house (38.6%), inside the house (25.1%), and near the water source (7.2%). Kachia showed the highest presence of feces around the households (63.2%), and all the LGAs showed high presence inside the households. Cow dung and animal excreta (42.3%) and children's feces (26.3%) were also seen around the premises. However, water sources were kept fairly free from the feces. Traditional pit toilets (89.5%) were the most observed. The observed features of the toilets are as follows: small enough hole (34.5%), adequate privacy (25.5%), safe floor (35.5%), presence of slab (16.1%), and having superstructure (23.1%). Locations of the toilets were mostly outside the compound (56.0%). In terms of the indicators for the current usage of the toilet, the following results were obtained: clear paths leading to it (50.4%), cleanliness (41.2%), being free of smell (23.4%), and being free of flies (22.0%). Hand wash facilities were located inside the house (21.7%) or within walking distance (11.5%) and next to the toilet (18.6%).

## 4. Discussion

### 4.1. Sources of Water for Drinking

Access to safe water supply is integral to health and survival [1]. In the study area, there is virtually total absence of improved *source of water*; thus, communities extensively utilize surface water and unprotected wells for drinking which can be infectious [12]; this is similar to many KAP surveys in developing areas [32, 40] including a recent counterpart study in Kaduna [41], Nigeria, where the majority of surveyed communities utilized polluted water sources due to lack of adequate clean water sources. However KAP studies by Pradhan et al. [42] and Hothur et al. [20] depict otherwise where almost 73.6% of the households were consuming water from improved source of water; this disparity is not unconnected to lack of effective water policy and governmental commitments in the respective study locations; efficient water policy can be helpful extensively towards mitigating the high WASH related mortality and morbidity rate in Nigeria [13].

### 4.2. Home Water Treatment Methods and Practices

Household water treatment practices can improve dramatically microbial drinking water quality and prevent diseases [43]; it is found to reduce rate of diarrhea infection among children [31] and shown to decrease level of cholera outbreak and disease transmission among people [44]. In the study area communities' level of *home water treatment practices* was considerably low as 54.8% do not treat water particularly using efficient methods such as chlorination. The absence of home treatment practices is often consistent in many communities across developing countries in the world as shown by many studies [28, 40, 45–47], being more pathetic in the rural areas [48, 49]; it is perceived unimportant by the rural dwellers due to lack of education and awareness on WASH, which has continued to impact negatively on their health status.

### 4.3. Storage System Practices, Handling, and Knowledge of Quality Drinking Water

Appropriate use of *storage vessels and handling attitudes* are vital to maintaining quality drinking water and preventing waterborne diseases at households [50–52]; uncovered water storage containers and those with wide openings make water susceptible to contamination; frequent cleaning of containers is essential in mitigating household water bacterial recontamination [33], and it is highly recommended as part of sanity that cups used to retrieve water from storage containers should have ladles or handles [46], to avoid unclean hands dipped into water in the process of fetching, and be kept on clean surface [40] or hung after usage. In the study area, different storage systems used are safe for storing water and are fairly covered and cleaned periodically; this is similar to findings by Reddy et al. [46], Pradhan et al. [42], and Ssemugabo et al. [53]. However the study population lacks knowledge of quality drinking water as many responded that quality water means visually clear water. This understanding affects home water treatment practices, consequently subjecting communities to diarrhea and waterborne diseases [31]. Contrarily, some similar studies show that water consumers are knowledgeable about safe drinking water [53].

### 4.4. Excreta Disposal Practices

The lack of *improved toilets facilities* was a huge concern in the study areas, resulting in open defecation being a common practice especially by children at households, which is a major health and social burden for the community at large [54]; this corresponds to various findings in developing countries [30, 46]. Nonetheless, the respondents have positive perception regarding good toilet facilities and are willing to stop open defecation; however, it was reported that unavailability of improved toilets was due to lack of financial capability. Toyobo et al. [55] and Miner et al. [15] reported similar scenarios, where communities suffer WASH challenges due to lack of fund. Livelihood empowerment and poverty eradication in the rural areas are vital to sustainable toilet facilities in the area.

### 4.5. Personal and Environmental Hygiene

In the study area adequate knowledge of *personal hygiene* led many to the good practices of using water and soap for cleaning hands after defecation, which is essential in preventing diseases [31], similar to studies by Orimoleye et al. [30] in Ibadan and Miner et al. [15] in Jos, Nigeria; practices of *environmental hygiene* were also fairly good as children feces were majorly perceived as harmful and were disposed in a toilet facility as reported by 63% of the respondents. However increased education is essential at critical times as children feces were observed in many households (38.6%) during inspection.

### 4.6. Waterborne and Water Related Diseases

Relating to the KAP on WASH in the study area, *waterborne diseases* of diarrhea, malaria, and dysentery seem to be prominent in the area; dysentery and diarrhea were more common among children under 5, relating to the findings by Yaya et al. [2] and Prüss-Ustün et al. [5] where leading cause of death under the age 5 in developing countries was diarrhea. Generally, the disease prevalence was low among the communities.

### 4.7. Constraints, Challenges, and Strength of the Research

During the research exercise, we were faced with some constraints and challenges which can affect the quality of the exercise which are lack of efficient means of transportation, poor accessibility to some communities, noncooperative attitude of some few respondents due to bulky nature of the questionnaire, and inadequate time for the exercise; however, the strength is the general cooperation of the community and stakeholders.

## 5. Conclusion and Recommendations

The deficiency in knowledge, poor attitudes, and lack of practices of WASH, particularly with regard to home water treatment, use of unsafe water sources, and open defecation, are routes of exposure to waterborne infections associated with the study area. Effectiveness of WASH does not depend on facilities provision alone. Therefore, WASH education is fundamental for promoting good practices and behavior towards WASH in order to protect public health. The study further suggests investigation regarding personal and environmental hygiene practices and the related disease implication in the study area.

## Figures and Tables

**Table 1:** Sample size and number of selected IYS LGAs in Kaduna state.

*S*/*N*	LGA	Community	Sample size/LGA
1	Chikun	Gwagwada	236
2	Kajuru	M/Kajuru	71
3	Soba	Soba	188
4	Kachia	Kachia (Gumel)	156
5	Z/Kataf	Gukwu	203

Total			854

**Table 2:** Characteristics of the communities in Kaduna state.

Variable	Chikun (236)	Kachia (156)	Kajuru (71)	Soba (188)	Zango/K (203)	Total (854)
Gender						
Male	86.6	73.5	90.4	98.9	75.1	84.5
Female	13.4	26.5	9.6	1.1	24.9	15.5

Residency in the household						
Less than 1 year	0.4	0.6	0.0	0.0	1.0	0.5
1–10 years	12.2	16.1	23.3	13.3	7.8	13.0
Above 10 years	84.5	83.2	76.7	86.7	75.1	81.8
Always	2.9	0.0	0.0	0.0	16.1	4.7

Age						
Below 30 years	21.4	13.5	13.7	13.3	17.6	16.6
30–50 years	50.0	47.1	61.6	47.3	40.5	47.6
Above 50 years	27.3	38.7	24.7	38.8	27.8	31.8

Marital status						
Single	2.1	4.5	9.6	6.9	2.0	4.2
Married	91.2	84.5	82.2	91.0	89.8	88.8
Divorced	1.3	1.9	0.0	1.1	0.5	1.0
Widowed	5.5	9.0	8.2	1.1	7.8	5.9

Ever attended school						
Yes	61.8	86.5	90.4	99.5	75.6	80.2
No	38.2	13.5	9.6	0.5	24.4	19.8

Highest school attended						
Quranic school	45.4	11.0	11.0	66.5	25.4	36.1
Basic literacy	1.3	4.5	1.4	3.2	6.8	3.6
Primary school	25.2	21.3	21.9	10.1	22.9	20.4
Vocational school	0.8	0.0	4.1	1.1	2.9	1.5
Secondary school	17.2	37.4	34.2	10.6	32.2	24.4
Postsecondary school	10.1	25.8	27.4	8.5	9.8	14.0

Religion						
Christianity	66.8	91.6	69.9	0.5	93.7	63.4
Islam	28.2	2.6	28.8	96.3	3.4	32.6
Others	5.0	5.8	1.4	3.2	2.9	4.0

Occupation						
Student	3.8	4.5	4.1	2.7	2.0	3.3
Housewife	2.5	3.9	4.1	1.1	10.2	4.4
Retired/pensioner	4.2	9.7	0.0	1.6	1.0	3.5
Farmer	46.6	38.1	35.6	60.6	62.0	50.9
Private employment	2.9	9.0	5.5	6.9	1.0	4.7
Self-employed	13.0	5.2	12.3	5.3	4.9	7.9
Civil servant	17.2	21.3	31.5	16.0	9.8	17.1
Others	4.2	3.9	2.7	1.6	2.4	3.0

**Table 3:** Sources of water for drinking and other domestic purposes (expressed as %).

Feature	Chikun	Kachia	Kajuru	Soba	Zango/K	Total
Sources of water for households						
Motorized borehole	0.4	0.0	0.2	2.1	0.0	0.8
Hand pump borehole	0.4	1.9	45.2	21.8	5.4	10.4
Protected dug well with hand pump	2.5	0.0	6.8	2.1	0.0	1.7
Protected hand dug well	39.5	2.6	61.6	69.1	18.5	36.2
Unprotected hand dug well	49.6	92.9	11.0	21.3	36.6	44.8
Developed spring	1.3	0.6	0.0	0.5	0.5	0.7
Undeveloped spring	14.3	1.3	0.0	1.1	2.0	4.9
Rain water harvesting	26.0	85.8	17.8	36.2	63.9	54.2
Bottled water	0.0	1.3	0.0	0.5	0.0	0.3
Sachet (pure) water	0.0	0.6	2.7	5.3	0.5	1.6
Tanker water vendor	0.4	5.2	0.0	2.1	0.5	1.6
Truck water vendors	0.4	78.7	0.0	26.1	0.5	20.1
Surface water (river/pond/lake)	37.4	89.0	30.1	3.7	95.1	52.5
Others	0.0	1.3	0.0	1.6	0.5	0.7

Main source of drinking water during dry season						
Motorized borehole	0.0	0.0	0.0	1.6	0.0	0.3
Hand pump borehole	0.4	0.6	45.2	20.7	2.0	9.1
Protected dug well with hand pump	1.3	0.0	11.0	2.7	0.5	2.0
Protected hand dug well	35.7	2.6	39.7	68.1	12.7	31.7
Unprotected hand dug well	34.0	91.6	11.0	16.0	24.4	36.2
Developed spring	0.8	0.0	1.4	0.0	0.5	0.5
Undeveloped spring	12.2	0.0	2.7	0.5	0.5	3.8
Rain water harvesting	8.8	20.0	2.7	12.8	6.8	10.7
Bottled water	0.4	1.3	0.0	0.5	0.0	0.5
Sachet (pure) water	0.4	9.7	1.4	5.3	1.0	3.4
Tanker water vendor	0.4	30.3	0.0	4.3	1.0	6.8
Truck water vendors	1.3	60.6	0.0	31.4	0.0	18.2
Surface water (river/pond/lake)	35.7	89.7	21.9	3.7	97.1	51.9
Others	0.0	0.6	0.0	3.7	0.0	0.9

Main source of drinking water during wet season						
Motorized borehole	0.0	0.0	0.0	1.6	0.0	0.3
Hand pump borehole	0.0	1.3	34.2	12.2	5.4	7.1
Protected dug well with hand pump	1.7	0.0	4.1	2.7	0.0	1.4
Protected hand dug well	13.4	2.6	60.3	62.8	13.2	26.2
Unprotected hand dug well	36.6	92.3	6.8	9.6	34.6	37.7
Developed spring	0.0	0.0	0.0	0.0	0.5	0.1
Undeveloped spring	3.4	1.9	0.0	0.5	0.5	1.5
Rain water harvesting	72.7	87.7	42.5	55.3	85.9	72.2
Bottled water	1.7	1.3	0.0	0.5	0.0	0.8
Sachet (pure) water	0.4	1.9	0.0	1.1	0.0	0.7
Tanker water vendor	0.0	0.6	0.01.6	1.6	0.5	0.6
Truck water vendors	0.0	13.5	0.0	27.7	1.0	8.7
Surface water (river/pond/lake)	13.4	14.8	13.7	0.5	83.9	27.7
Others	0.0	0.6	0.0	0.0	0.0	0.1

Main source of water for other domestic purposes						
Motorized borehole	0.0	0.6	2.7	1.6	0.0	0.7
Hand pump borehole	0.0	1.3	37.0	13.8	6.3	7.9
Protected dug well with hand pump	2.5	0.0	0.0	2.1	0.0	1.2
Protected hand dug well	36.6	3.9	64.4	67.6	16.1	34.9
Unprotected hand dug well	44.5	85.2	8.2	16.0	30.2	39.1
Developed spring	0.4	0.0	0.0	0.0	0.0	0.1
Undeveloped spring	13.9	0.6	0.0	1.1	1.0	4.4
Rain water harvesting	52.5	70.3	11.0	21.3	42.0	42.8
Tanker water vendor	0.4	1.3	0.0	1.6	0.0	0.7
Truck water vendors	0.0	69.0	0.0	26.6	1.0	18.5
Surface water (river/pond/lake)	30.3	79.4	23.3	3.7	93.7	47.8
Others	0.0	1.3	0.0	1.6	0.5	0.7

**Table 4:** Water treatment practice by communities.

Water treatment for safe drinking	Chikun	Kachia	Kajuru	Soba	Zango	Total%
Yes	30.3	16.8	38.4	56.4	65.9	42.7
No	67.2	83.2	60.3	43.1	33.7	56.2
Never	1.7	0.0	1.4	0.0	0.5	0.7

**Table 5:** Handling and storage of household drinking water and knowledge of qualities of safe drinking water.

Features	Chikun	Kachia	Kajuru	Soba	Zango	Total%
Containers/fetching methods						
Open container	8.8	11.0	4.1	26.1	19.5	15.1
Covered plastic container	29.8	72.9	39.7	46.8	56.1	48.4
Clay pots with cover	53.4	65.8	58.9	41.5	56.1	54.1
Clay pots without cover	12.6	7.7	1.4	8.5	20.0	11.6
Iron bucket containers without cover	0.4	5.2	0.0	18.1	1.5	5.4
Plastic buckets with tap	2.5	24.5	11.0	11.7	2.4	9.2
Basins without cover	1.3	2.6	0.0	4.3	1.5	2.1
Others	4.6	0.0	0.0	0.5	0.0	1.4

Item used in fetching drinking water from storage facility						
Cup with handle	81.1	81.9	91.8	85.6	88.3	84.9
Cup without handle	5.5	5.8	2.7	4.8	4.4	4.9
Calabash	13.0	11.0	1.4	6.4	4.9	8.3
Bowl	0.4	1.3	4.1	3.2	2.4	2.0

Place where item for fetching drinking water is kept						
On the storage container	72.3	47.1	49.3	46.8	68.8	59.4
In a basket/shelf	14.7	33.5	39.7	16.5	22.4	22.5
On the floor	4.2	1.3	4.1	10.1	6.8	5.6
Hanging	8.8	18.1	6.8	26.6	2.0	12.6

Frequency of cleaning of storage container						
Daily	55.5	61.3	87.7	84.0	85.4	72.6
Weekly	26.9	23.2	1.4	10.1	4.4	15.0
Monthly	0.4	3.2	0.0	0.0	0.5	0.8
When dirty	17.2	12.3	11.0	5.9	9.8	11.5

**Table 6:** Excreta disposal methods and practices.

Feature	Chikun	Kachia	Kajuru	Soba	Zango	Total
Types of excretal disposal facilities in community						
Open defecation	48.3	3.2	41.1	10.1	47.3	41.4
Digging, defecating, and burying in soil	2.5	6.5	13.7	8.0	3.9	5.7
Traditional pit toilet	87.4	90.3	93.2	76.6	91.2	76.5
Improved pit toilets	1.7	22.6	23.3	6.4	12.2	10.8
VIP toilets	0.0	47.1	8.2	3.7	0.0	10.0
Pour flush toilets	0.0	10.3	20.5	11.7	1.0	6.4
Water closet toilets	0.0	8.4	8.2	1.1	0.0	2.4
Others	0.0	0.0	0.0	0.5	0.0	0.1

Types of excretal disposal facilities in households						
Open defecation	2.8	0.0	9.6	8.0	29.8	22.8
Digging, defecating, and burying in soil	0.4	0.6	1.4	7.4	0.5	2.1
Traditional pit toilet	72.7	85.8	74.0	76.1	62.0	65.7
Improved pit toilets	2.5	0.6	12.3	6.4	9.8	5.9
VIP toilets	0.0	3.9	2.7	2.1	0.0	1.4
Pour flush toilets	0.0	0.0	1.4	1.6	0.0	1.5
Water closet toilets	0.4	0.6	1.4	0.5	0.0	0.5
Others	0.0	0.0	0.0	0.0	0.0	0.0

**Table 7:** Disposal of children's feces.

Feature	Chikun	Kachia	Kajuru	Soba	Zango/K	Total
Under-5 defecation						
Around the house	22.3	19.4	13.7	14.4	47.8	25.4
In the potty/chamber pot	34.0	0.6	37.0	26.1	26.3	24.7
In the toilet	22.3	67.7	17.8	31.4	6.3	28.3
In pampers	1.3	0.0	0.0	4.8	0.5	1.5
Within the compound	10.5	0.0	5.5	1.6	2.9	14.7
Others	9.2	12.3	16.4	21.3	16.1	14.7

Methods of disposal of children's feces						
Dropped into a toilet facility	59.7	83.9	72.6	81.9	30.2	63.0
Eaten by dogs	3.8	6.5	2.7	0.5	32.7	10.4
Buried in the soil	9.7	3.9	1.4	5.3	8.3	6.6
Thrown into the bush	18.1	1.3	6.8	2.1	42.0	16.3
Disposed with solid waste	3.4	0.6	2.7	1.1	0.5	1.6
Nothing/left there	0.4	0.6	0.0	1.1	8.8	2.6

**Table 8:** Perception of a good toilet, type of toilet, and preferred ownership.

Feature	Chikun	Kachia	Kajuru	Soba	Zango	Total (%)
Perception of a good toilet						
Privacy	58.4	52.3	41.1	26.6	48.3	46.4
Safety	31.1	36.1	26.0	34.0	34.6	33.1
Preventing diseases	21.8	66.5	41.1	58.5	48.8	46.0
Easy to use	8.0	26.5	19.2	22.9	18.0	17.9
Well covered and clean	9.7	11.6	47.9	5.3	29.8	17.1
Used by children on their own	7.1	16.1	1.4	2.1	11.2	8.1
Built close to the house	0.8	0.6	4.1	0.0	2.0	1.2
Others	0.0	0.0	2.7	0.5	0.0	0.3

**Table 9:** Personal hygiene practices.

Feature	Chikun	Kachia	Kajuru	Soba	Zango	Total (%)
Uses of soap						
Washing clothes	71.8	89.7	83.6	78.2	72.2	77.5
Taking bath	84.9	80.0	94.5	80.3	91.7	85.4
Bathing children	38.7	83.2	21.9	60.6	36.5	49.4
Washing child's bottom	2.9	36.1	12.3	28.7	16.1	18.5
Washing children's hands	1.3	36.1	11.0	38.8	16.6	20.3
Washing hands after defecating	5.9	56.8	28.8	47.9	27.3	31.3
Washing hands after cleaning child	1.3	29.0	12.3	28.7	14.6	16.4
Washing hands before feeding child	1.7	34.8	16.4	38.4	15.1	20.4
Washing hands before preparing food	0.4	40.0	19.2	46.3	18.0	23.4
Washing hands before eating	5.9	53.5	47.9	50.0	33.7	34.3
Others	0.4	5.8	1.4	3.7	0.0	2.1

Important time to wash hands						
Before meal	91.6	71.0	75.3	87.2	89.8	85.1
After meal	68.5	65.8	65.8	71.3	89.8	73.5
After defecation	26.9	76.8	38.4	43.1	53.7	46.8
After cleaning the children feces	4.6	11.0	17.8	15.3	22.4	13.5
Others	0.4	15.5	13.7	5.9	2.0	5.8

Immediate practice after defecation						
Cleaning up	71.4	60.0	20.5	18.1	59.5	50.5
Going own way	1.7	1.3	2.7	6.4	2.9	3.0
Washing hands	26.9	38.7	76.7	75.5	37.6	46.4

Items for hand washing						
Water only	32.8	8.3	30.4	9.2	39.0	21.6
Water with soap	53.1	63.3	58.9	78.9	57.1	65.4
Water with ashes	0.0	0.0	3.6	9.2	0.0	3.8
Sand and water	0.0	0.0	3.6	21.8	2.6	8.8
Others	0.0	0.0	0.0	1.4	1.3	0.8

**Table 10:** Major problems of diseases perceived in communities and households.

Feature	Chikun	Kachia	Kajuru	Soba	Zango	Total (%)
Common diseases in community						
Malaria	81.9	98.1	91.8	88.3	88.3	88.6
Measles	41.6	42.6	60.3	61.7	58.5	51.8
Diarrhea/dysentery	40.3	24.5	17.8	21.3	47.3	33.1
Cholera	13.4	12.9	30.1	11.2	48.3	22.6
Yellow fever	8.4	60.6	13.7	28.2	30.2	27.8
Chicken pox	9.7	31.0	41.1	23.9	30.2	24.2
Meningitis	11.8	23.2	6.8	9.0	25.4	16.1
Typhoid	45.4	76.8	47.9	37.8	74.1	56.5
Guinea worm	0.0	0.0	0.0	0.0	0.0	0.0
Onchocerciasis	0.0	1.3	0.0	3.7	4.4	2.1
Trachoma	0.0	3.2	0.0	3.7	1.0	1.6
Schistosomiasis	0.0	0.0	0.0	2.7	1.0	0.8
Worm infestations	0.8	6.5	0.0	3.2	13.2	5.2
Scabies	0.4	4.5	1.4	3.2	7.8	3.6
Others	0.4	0.6	1.4	2.1	2.4	1.4

Major childhood diseases						
Malaria	70.6	85.2	78.1	76.6	79.0	77.2
Measles	67.2	58.1	64.4	71.3	56.1	63.6
Diarrhea/dysentery	39.5	12.9	13.7	21.3	40.5	28.8
Cholera	8.4	5.2	11.0	13.8	13.8	15.8
Yellow fever	4.2	54.2	23.3	28.7	26.3	25.5
Chicken pox	10.5	25.8	52.1	18.1	27.8	22.6
Meningitis	10.1	32.3	1.4	7.4	17.6	14.6
Typhoid	24.8	24.5	8.2	24.5	46.8	28.5
Guinea worm	0.0	0.0	0.0	0.0	0.0	0.0
Onchocerciasis	0.0	0.0	1.4	2.7	1.5	1.0
Trachoma	0.4	0.0	0.0	2.7	0.5	0.8
Schistosomiasis	0.0	0.0	0.0	2.1	0.5	0.6
Worm infestations	0.4	7.1	0.0	2.1	10.7	4.4
Scabies	0.0	1.9	2.7	1.6	9.3	3.1
Others	0.0	1.3	2.7	1.1	2.9	1.4

**Table 11:** Structured observations.

Feature	Chikun	Kachia	Kajuru	Soba	Zango/k	Total
Evidence of feces around the premises						
Inside the house	30.3	36.1	21.9	29.8	7.8	25.1
Outside/around the house	9.7	63.2	31.5	19.1	74.1	38.6
Near the water source	3.8	14.8	4.1	9.0	4.9	7.2

Observations on the feces around the premises						
Infants/young children's feces	34.5	26.5	15.1	22.3	24.4	26.3
Adults' feces	5.5	25.2	4.1	11.2	12.7	11.9
Cow dung and other animal feces	15.5	63.9	56.2	24.5	68.3	42.3

Type of toilet observed						
Digging, defecating, and burying in soil	1.3	1.3	1.4	6.9	4.9	3.4
Traditional pit toilet	97.5	91.0	84.9	83.0	86.8	89.5
Improved pit toilets	0.8	3.2	8.2	4.3	7.3	4.2
VIP toilets	0.0	4.5	2.7	2.7	0.0	1.6
Others	0.0	0.0	0.0	0.0	0.5	1.2

Features of the toilet in the household (if available)						
Having superstructure	21.0	25.2	35.6	30.9	12.2	23.1
Safe floor	8.8	72.3	74.0	45.3	16.1	35.5
Having a slab	27.3	12.3	23.3	16.0	3.4	16.1
Small enough hole	17.2	55.5	58.9	35.6	28.8	34.5
Adequate privacy	18.5	28.4	60.3	30.3	14.6	25.5

Location of toilet						
Inside the compound	48.7	29.7	52.1	75.0	18.0	44.0
Outside the compound	51.3	70.3	47.9	25.0	82.0	56.0

Toilet in current use						
Clear path leading to it	45.8	75.5	78.1	51.1	26.3	50.4
Being clean	18.9	72.3	75.3	57.4	16.6	41.2
Being reasonably free of smell	9.7	38.1	64.4	30.9	6.8	23.4
Being reasonably free of flies	9.2	41.3	58.9	24.5	6.8	22.0
Cleansing materials	2.5	63.9	42.5	18.1	2.9	20.5
Presence of water in the vicinity	1.3	48.4	39.7	22.9	2.9	18.2
Presence of ash in the vicinity	0.8	28.4	46.6	13.8	1.5	12.7
Any other evidence of use	8.8	41.3	43.8	18.6	3.4	18.5

Presence of hand washing facility						
Next to the toilet	2.1	45.2	19.2	33.5	3.9	18.6
Within walking distance	0.4	30.3	15.1	16.5	4.4	11.5
Inside the house	0.4	29.0	30.1	21.8	37.6	21.7

Observing the presence of the following						
Storage container	22.7	26.5	32.9	41.5	46.8	34.1
Separate bowl/cup to fetch water	5.0	19.4	34.2	43.1	43.9	27.7

## Data Availability

The data used to support the findings of this study are available from the corresponding author upon request.
